# Impact of asthma in Europe: A comparison of web search data in 21 European countries

**DOI:** 10.1016/j.waojou.2023.100805

**Published:** 2023-08-02

**Authors:** Hannah Wecker, Linda Tizek, Stefanie Ziehfreund, Alphina Kain, Claudia Traidl-Hoffmann, Gregor S. Zimmermann, Emanuele Scala, Jesper Elberling, Anaïs Doll, Michael J. Boffa, Lea Schmidt, Mariusz Sikora, Tiago Torres, Natalia Ballardini, Pavel V. Chernyshov, Jeroen Buters, Tilo Biedermann, Alexander Zink

**Affiliations:** a Technical University of Munich, School of Medicine, Department of Dermatology and Allergy, Munich, Germany; b Environmental Medicine, Faculty of Medicine, University of Augsburg, Augsburg, Germany; c Institute of Environmental Medicine, German Research Center for Environmental Health, Helmholtz Zentrum München, Neuherberg, Germany; d Department of Respiratory Medicine, InnKlinikum, Academic Hospital of the Technical University of Munich, Muehldorf am Inn, Germany; e Department of Internal Medicine I, School of Medicine, University Hospital Rechts der Isar, Technical University of Munich, Munich, Germany; f Department of Dermatology and Venereology, Medical Center—University of Freiburg, Faculty of Medicine, University of Freiburg, Freiburg, Germany; g Division of Dermatology and Venereology, Department of Medicine Solna and Center for Molecular Medicine, Karolinska Institutet, Stockholm, Sweden; h Depart of Allergy, Herlev and Gentofte Hospital, Copenhagen, Denmark; i Dept of Clinical Medicine, University of Copenhagen, Copenhagen, Denmark; j Department of Dermatology, Mater Dei Hospital, Msida, Malta; k National Institute of Geriatrics, Rheumatology and Rehabilitation, Warsaw, Poland; l Department of Dermatology, Centro Hospitalar Universitário do Porto, University of Porto, Porto, Portugal; m Institute of Environmental Medicine, Karolinska Institutet, Stockholm, Sweden; n Sachs' Children and Youth Hospital, Södersjukhuset, Stockholm, Sweden; o Department of Clinical Science and Education Södersjukhuset, Karolinska Institutet, Stockholm, Sweden; p Department of Dermatology and Venereology, National Medical University, Kiev, Ukraine; q Center of Allergy & Environment (ZAUM), Member of the German Center for Lung Research (DZL), Technical University and Helmholtz Center Munich, Munich, Germany; r Division of Dermatology and Venereology, Department of Medicine Solna, Karolinska Institutet, Stockholm, Sweden

**Keywords:** Asthma, COVID-19, Infodemiology, Pollen, Web search

## Abstract

**Background:**

Asthma is a chronic inflammatory disorder of the airways and one of the most important non-communicable diseases worldwide. Analyzing crowdsourced data can help understand public interest and unmet needs as well as potential factors influencing search behavior.

**Objective:**

The study aimed to investigate asthma-related web search data in Europe to identify possible regional and seasonal variations and to assess public interest.

**Methods:**

Google Ads Keyword Planner was used to measure search volume for search terms related to *asthma*, *allergic asthma*, and *bronchial asthma* in 21 European countries between January 2018 and December 2021. The top 10 keywords of each country were categorized qualitatively. Search volume per 100 000 inhabitants was descriptively assessed in terms of regional and seasonal trends. Spearman correlations between search volume and pollen concentration as well as coronavirus disease (COVID-19) cases were investigated.

**Results:**

The median search volume per 100 000 inhabitants for *asthma* and *allergic asthma* was highest in Northern and Western Europe, while the highest search volume for *bronchial asthma* was observed in Western and Eastern regions. A seasonal trend was identified for all search terms and in all regions. Correlations were found between search frequency and pollen load and search behavior and COVID-19 cases. Overall, Europeans were most interested in the diseases in general, their treatment options, and symptoms.

**Conclusion:**

These results highlighted the need for reliable and region-specific information about the disease and for public campaigns to improve asthma control. The study also emphasizes the importance of using crowdsourced data for a more encompassing overview beyond conventional healthcare data.

## Introduction

Asthma, also known as asthma bronchiale or bronchial asthma, is a chronic inflammatory disorder of the airways that causes recurrent episodes of wheezing, breathlessness, chest tightness, and coughing with associated airflow obstruction.^1^ The etiology is not known, but genetics and risk factors such as respiratory infections and airborne environmental exposures play an important role.^2^^,^^3^

The World Health Organization (WHO) declared asthma as one of the major non-communicable diseases worldwide, affecting both children and adults. In 2019, an estimated 262 million people around the world suffered from asthma, causing 455 000 deaths.^4^ There are geographic variations in asthma prevalence, with a higher number of asthma patients observed in high-income countries.^5^ Overall Europe, the prevalence of asthma ranges from 5.1% to 8.2% in adults,^5^, ^6^, ^7^ although large differences exist between European countries, with 1.3% in Bosnia and 17.6% in the United Kingdom (UK). The prevalence of asthma and allergic asthma is increasing;^2^^,^^8^ for example, in Sweden, a significant increase in the prevalence of asthma was observed from 8.4% in 1996 to 10.9% in 2016.^8^ Asthma can be well controlled with guideline-based therapy.^9^ Nevertheless, asthma is undertreated and poorly controlled in Europe and is therefore an important public health problem that urgently needs further investigation.^10^^,^^11^

In recent years, more people have searched for health information online.^12^ Due to growing internet use, analyzing web-based data on public health issues, also known as infodemiology, is increasingly relevant for understanding the interest and needs of a population and can help predict disease outbreaks and hospitalization rates.^13^, ^14^, ^15^, ^16^ Since Google has the highest market share in all of Europe with more than 90%,^17^ its search engine queries reflect the online interests of the European population. Previous research has examined online search queries for asthma, mainly focusing on allergic rhinitis and pollen season^18^, ^19^, ^20^ and using the relative number of searches.^14^^,^^21^^,^^22^ Studies comparing the absolute number of searches for different asthma search terms in European countries to investigate possible unmet needs in the European population are still lacking.

The most common asthma phenotype is allergic asthma, which is asthma associated with sensitization to inhalant allergens and often related to a history of eczema and allergic rhinitis.^23^ Since allergic asthma is triggered by aeroallergens, especially birch and grass pollen, asthma attacks and exacerbations occur seasonally depending on the seasonal pollen counts.^23^, ^24^, ^25^, ^26^ These seasonal patterns and associations have been demonstrated in the literature for allergy-related web searches,^18^, ^19^, ^20^ while no association with asthma-related searches has been found in European countries.^18^^,^^19^ However, such studies are pending for allergic asthma.

Some symptoms of coronavirus disease (COVID-19) strongly resemble asthma symptoms and are therefore very difficult to distinguish.^21^ At the same time, both asthma and chronic obstructive pulmonary disease (COPD) have an impact on disease expression and outcome in patients with COVID-19.^1^^,^^29^ Consequently, the association of asthma and COVID-19 has received growing interest, and researchers suspect that the appearance of asthma symptoms may have masked actual COVID-19 infections based on a massive increase in asthma-related web searches at the beginning of the pandemic.^21^^,^^30^ Studies of the association between COVID-19 cases and internet searches are limited to the outbreak of COVID-19 and the subsequent course has not yet been studied in detail.

### Objectives

The aim of the study was to gain insight into asthma-related search behavior across Europe to identify possible regional and seasonal variations and to determine public interest and unmet needs in asthma. Additionally, web search queries were compared with pollen levels and COVID-19 incidences to explain potential search patterns.

## Methods

### Search volume data

Google Ads Keyword Planner was used to measure online search volume (the number of searches for a topic or search term; SV) for search terms related to *asthma, allergic asthma*, and *bronchial asthma*. This tool was originally developed to optimize advertising, but it is now also used in research.^13^, ^14^, ^15^, ^16^ Search terms or phrases are entered into the tool which then finds associated keywords. The outcome of Google Ads Keyword Planner is an estimate of the monthly number of search queries for each keyword in the last 48 months, the maximum possible period.

In this retrospective study, the monthly SV was analyzed from January 2018 to December 2021 in 21 European countries (Austria, Bosnia, Croatia, Czech, Denmark, France, Germany, Greece, Hungary, Ireland, Italy, Malta, Netherlands, Poland, Portugal, Romania, Serbia, Spain, Sweden, United Kingdom, and Ukraine), covering a population of 527 million Europeans,^31^ about 81% of whom have internet access.^12^ The selection of these 21 countries was based on previous collaborations and the authors’ network of native speakers and researchers in this field to ensure the correct translation of the keywords. Due to country-specific differences in searching for the same disease because of different linguistic expressions,^19^ this study examined the 3 terms *asthma*, *allergic asthma*, and *bronchial asthma* across all countries. For each country, the translated terms were entered in Google Ads Keyword Planner (Table 1). Region and language settings had been set to provide data only from users in the aforementioned countries and in their native or main spoken language. For Malta, the English language was used since Google AdWords Keyword Planner does not include Maltese as a selection option. Translation of the search terms and the back translation of the 10 most frequent keywords were performed by native speakers and/or researchers specialized in the field of allergic asthma in their countries of origin. For 3 countries (Greece, Spain, and Czech), translation was done with DeepL. The 21 countries were categorized geographically into 5 European groups based on the United Nations (UN) classification:^32^ Northern (Denmark, Ireland, Sweden, and United Kingdom), Eastern (Czech, Hungary, Poland, Romania, and Ukraine), Western (Austria, France, Germany, and the Netherlands), Southern (Italy, Malta, Portugal, and Spain), and Southeastern Europe (Bosnia, Croatia, Greece, and Serbia).

**Table 1:** Population size in 2019, Google search terms in the original language, and the corresponding number of keywords related to the search terms for each European country examined in this study.

		Asthma		Allergic asthma		Bronchial asthma	
Country	Population size in 2019	Original language	No. keywords	Original language	No. keywords	Original language	No. keywords
Austria	8 858 775	asthma	985	allergisches asthma	315	asthma bronchiales	335
Bosnia	3 492 018	astma	126	alergija astma	20	bronhijalna astma	10
Croatia	407 624 6	astma	135	astma alergija	20	bronhijalna astma	10
Czech	10 649 800	astma	80	alergické astma	7	bronchiální astma	2
Denmark	5 806 081	astma	238	allergisk astma	3	bronkial astma	1
France	67 290 471	asthme	1274	asthme allergique	164	asthme bronchique	17
Germany	83 019 213	asthma	990	allergisches asthma	318	asthma bronchiales	335
Greece	10 724 599	άσθμα	265	αλλεργικό άσθμα	35	βρογχικο ασθμα	46
Hungary	9 772 756	asztma	177	allergiás asztma	16	asthma bronchiale	15
Ireland	4 904 240	asthma	760	allergic asthma	270	bronchial asthma	94
Italy	59 816 673	asma	737	asma allergica	207	asma bronchiale	107
Malta^a^	493 559	asthma	710	Allergic asthma	110	bronchial asthma	36
Netherlands	17 282 163	astma	682	allergische astma	54	bronchiale astma	23
Poland	37 972 812	astma	375	astma alergiczna	34	astma oskrzelowa	297
Portugal	10 276 617	asma	568	asma alérgica	31	Asma bronquica	28
Romania	19 414 458	astm	353	astm alergic	47	astm bronsic	255
Serbia	6 963 764	astma	94	astma alergija	7	Bronhijalna astma	14
Spain	46 937 060	asma	859	asma alérgica	162	asma bronquial	160
Sweden	10 230 185	astma	368	allergisk astma	22	bronkialastma	1
UK	66 647 112	asthma	733	allergic asthma	356	bronchial asthma	146
Ukraine	41 983 564	астма	37	алергічна астма	3	бронхіальна астма	66

No., number of; UK, United Kingdom.

a The English language was used because Google AdWords Keyword Planner does not include the Maltese language as a selection option.

### Other data sources

Daily pollen concentrations from January 2019 to December 2021 were obtained from a monitoring station (Biederstein) in Munich as regional reference for pollen data. Since birch and grass pollen in particular are associated with asthma symptoms,^23^^,^^24^^,^^26^ these pollen species were investigated in this study. The pollen concentrations were summarized for each species as the monthly pollen load.

The WHO provides daily data on the worldwide number of COVID-19 cases from January 2020 onwards.^28^ For this study, data from January 2020 to December 2021 from the 21 countries examined were used. Data were first summarized on a monthly basis and then on a region-specific basis by forming the median for the corresponding countries.

Institutional review board approval and informed consent were not applicable for this study.

### Statistical analysis

For comparability between countries, monthly SV and COVID-19 data were calculated per 100 000 inhabitants (SV/100 000 inhabitants). The population size in 2019 was extracted from the Statistical Office of the European Union,^31^ since all examined countries provided information that year. The SV of the regions was summarized by the median of the countries. To investigate linear and seasonal trends, SV over time was considered as a time series. Analyses were performed descriptively and by decomposing the time series into its components: a trend, seasonal, and random component. Differences between regions and European seasons (spring: March to May; summer: June to August; fall: September to November; winter: December to February) were tested with Kruskal-Wallis tests and Friedman tests, respectively, as the SV in regions and seasons was not normally distributed. Afterwards, a Dunn's test or paired Wilcoxon signed-rank test with Bonferroni correction was used for post-hoc tests for pairwise comparisons. Pollen concentration, COVID-19 incidence, and SV data were compared descriptively after normalization and spearman correlations (r) were calculated. Due to the regional variations in pollen load, the relationship between SV and pollen concentration was only assessed for Western European regions. SV was reported with median and interquartile range (IQR).

The 10 most searched keywords in combination with *asthma*, *allergic asthma*, and *bronchial asthma* for each country were analyzed qualitatively and categorized by 2 researchers independently (HW, LT) according to whether they referred to the search term in general (eg, “asthma”), symptoms (eg, “asthma symptoms”), attacks (eg, “asthma attack”), treatment (eg, “inhaler”), natural/home remedies (eg, “allergic asthma home remedy”), information (eg, “asthma bronchiale forum”), triggers (eg, “hayfever asthma”), progression (eg, “bronchial asthma complications”), children (eg, “asthma in children”), and cats (eg, “asthma in cats”). Each keyword was classified into one category. The frequency of the categories was represented graphically between European regions in mosaic plots.

The level of significance was set at α = 0.05. Statistical analyses were performed using R version 4.0.4. (R Core Team, 2021) and the following R packages for mapping Europe “rnaturalearth”, “rnaturalearthdata”, and “rgeos”.

## Results

Overall, 46 104 750 searches for *asthma*-related keywords were recorded between January 2018 and December 2021, with 2 644 910 searches for *allergic asthma* and 7 706 270 for *bronchial asthma*. The highest median monthly SV/100 000 inhabitants was observed for *asthma* (median: 121.67, IQR: [78.67, 213.56]), followed by notably fewer searches for *bronchial asthma* (10.55 [5.39, 29.68]) and *allergic asthma* (8.28 [5.01, 12.99], Supplemental Table 1).

### Differences between countries

**Fig. 1:**
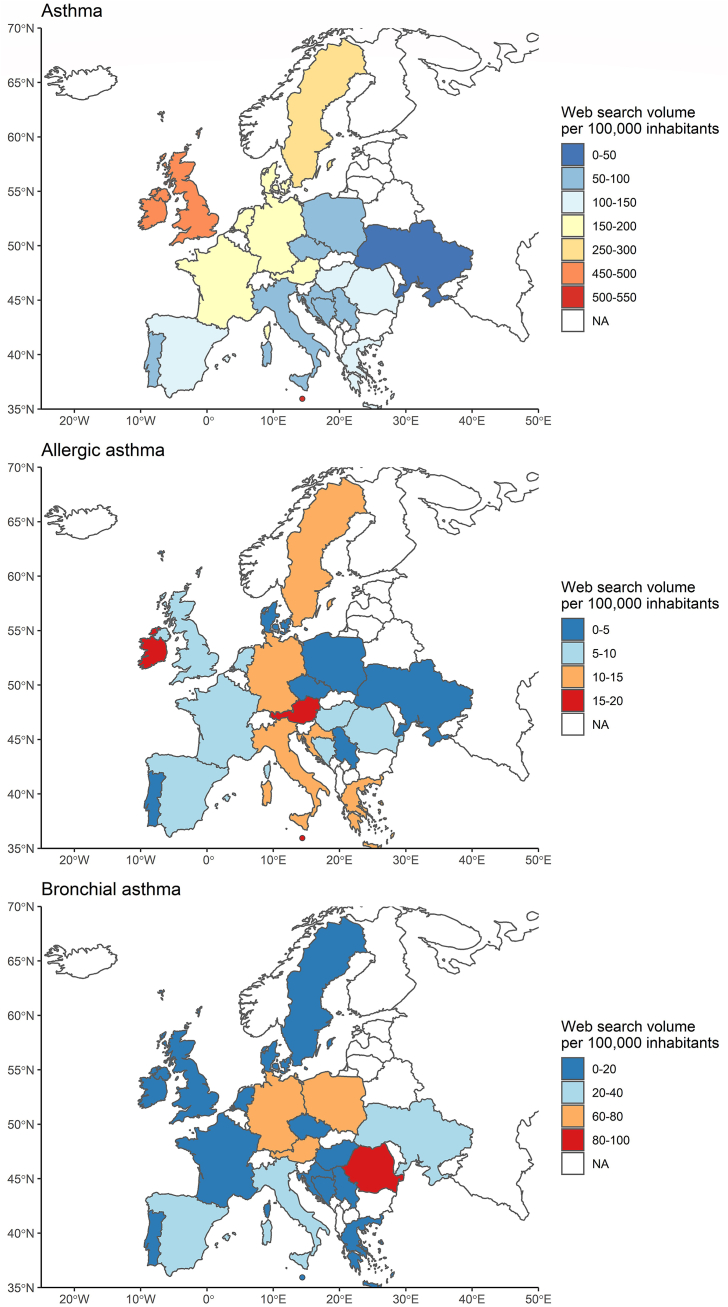
Median web search volume between 2018 and 2021 for each European country examined, with separate maps for the search terms *asthma, allergic asthma*, and *bronchial asthma*. Malta was displayed as an enlarged dot for better illustration.

The highest SV per month for *asthma*-related keywords was reported in Malta with 541.98 (480.69, 630.62) searches per 100 000 inhabitants, whereas less than 3% of searches were observed in Ukraine, the country with the lowest *asthma* SV/100 000 inhabitants (14.99 [11.79, 18.51]). The highest SV for *allergic asthma* was observed in Austria, with 19.36 (14.79, 24.3) searches per month. The lowest SV/100 000 inhabitants was observed in Ukraine, with 0.1 (0.07, 0.17) searches per month. With 83.21 (70, 97.41) monthly searches, Romania had the highest SV/100 000 inhabitants for *bronchial asthma*. In contrast, Denmark showed the lowest SV/100 000 inhabitants (0.17 [0.17, 0.17], Fig. 1, Supplemental Table 1, Supplemental Figure 1).

### Differences between regions

**Fig. 2:**
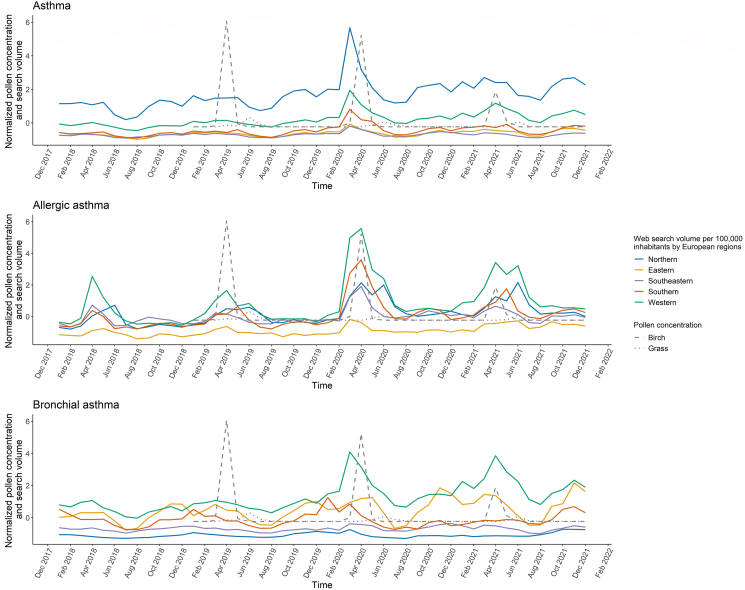
Time course of the normalized median web search volume per 100 000 inhabitants for search terms *asthma, allergic asthma*, and *bronchial asthma* between 2018 and 2021 (solid lines) separately for the 5 European regions and normalized pollen concentration of birch (dashed line) and grass (dotted line) from a Munich measuring station. European regions were defined geographically as Northern (Denmark, Ireland, Sweden, and United Kingdom), Eastern (Czech, Hungary, Poland, Romania, and Ukraine), Western (Austria, France, Germany, and the Netherlands), Southern (Italy, Malta, Portugal, and Spain), and Southeastern Europe (Bosnia, Croatia, Greece, and Serbia).

Regional differences were found for all search terms (p < 0.001, Supplemental Table 1, Fig. 2). Northern and Western European countries showed the highest monthly SV/100 000 inhabitants for *asthma* (Northern: 357.91 [314.61, 427.62]; 179.97 [155.67, 216.24], p = 0.007) and *allergic asthma* (Northern: 10.53 [7.62, 12.79]; Western: 12.59 [9.07, 16.06], p = 0.349). For *bronchial asthma*, however, the highest SV/100 000 inhabitants were reported for Western and Eastern Europe with 33.93 (29.19, 43.06) and 26.18 (20.53, 32.11) searches (p = 0.106), respectively. The lowest SV for *bronchial asthma* was reported in Northern Europe (4.43 [3.68, 5.79], p < 0.001). No differences were observed between the SV for *allergic asthma* in northern, southern, and southeastern countries in pairwise comparisons (p = 1.000), and fewer substantial differences were observed for queries related to *bronchial asthma* between Eastern and Southern Europe (p = 0.088).

### Time course

For *asthma*, all regions recorded a peak in March 2020, the start of the COVID-19 pandemic in Europe. At this time, the SV/100 000 inhabitants for *asthma* doubled in nearly all regions. No clear seasonal trend was observed, but searches decreased significantly in the European summer months (p < 0.001). In all regions, the SV/100 000 inhabitants increased over time. The SV/100 000 inhabitants for *allergic asthma* also showed a peak in March 2020 in all regions except for the north, where the highest SV of 22.32 searches per month was recorded in June 2021. The SV for *allergic asthma* showed a positive trend over time, with a higher SV/100 000 inhabitants being observed in spring (p < 0.001) and a lower SV in winter (0.001 < p ≤ 0.029), indicating a seasonal trend. For *bronchial asthma*, a decrease in SV/100 000 inhabitants was observed in all regions during the summer months (p < 0.001) after a peak in March, with most regions recording a second or third peak in the autumn or winter months. In Northern, Eastern, and Western Europe, SV/100 000 inhabitants increased over time, while in the other regions it leveled off over time (Supplemental Table 1, Fig. 2).

### Web search data and pollen

Comparing the monthly normalized SV/100 000 inhabitants of Western European countries and birch and grass pollen concentrations in Munich over time (Fig. 2), parallels were observed for all search terms, particularly in 2020 and 2021. Moderate to strong correlations were found between monthly SV and birch pollen (0.41 ≤ r ≤ 0.65, p ≤ 0.014), while negative associations were observed between grass pollen and SV for *asthma* (r = −0.30, p = 0.074) and *bronchial asthma* (r = −0.33, p = 0.048). Peaks in *allergic asthma* SV/100 000 inhabitants and Bavarian pollen concentration occurred simultaneously in April and June, and web searches in other European regions also reached their annual maximum during the 2 pollen seasons in Bavaria.

### Web search data and COVID-19 incidences

**Fig. 3:**
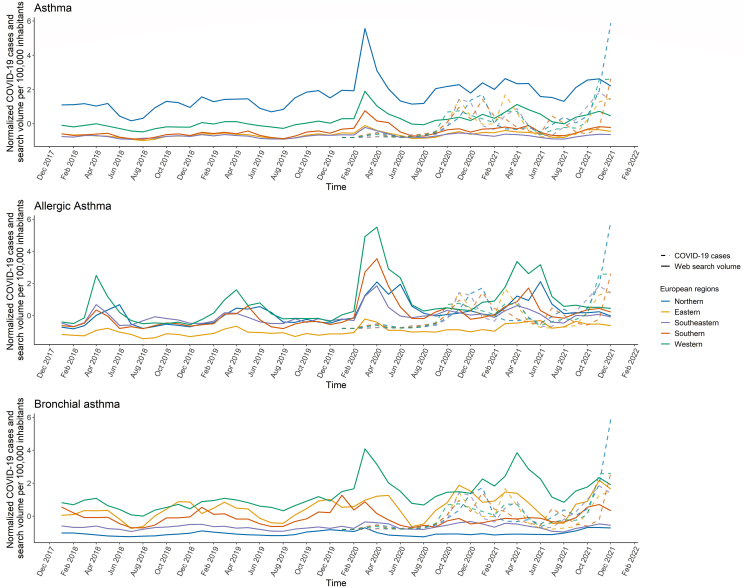
Time course of the normalized median web search volume per 100 000 inhabitants for search terms *asthma, allergic asthma,* and *bronchial asthma* between 2018 and 2021 (solid lines) and the normalized median number of COVID-19 cases between 2020 and 2021 (dashed lines) separately for the 5 European regions: Northern (Denmark, Ireland, Sweden, and United Kingdom), Eastern (Czech, Hungary, Poland, Romania, and Ukraine), Western (Austria, France, Germany, and the Netherlands), Southern (Italy, Malta, Portugal, and Spain), and Southeastern Europe (Bosnia, Croatia, Greece, and Serbia).

Comparing normalized SV/100 000 inhabitants and the number of COVID-19 cases over time (Fig. 3) demonstrated similarities for *asthma* and *bronchial asthma*. In particular, Northern (r = 0.31, p = 0.002), Eastern (r = 0.38, p < 0.001), and Western (r = 0.26, p = 0.010) European regions showed moderate correlations between *asthma* SV and COVID-19 incidences, while moderate to strong correlations were observed for *bronchial asthma* in almost all regions (Northern: r = 0.33, p = 0.001; Eastern: r = 0.64, p < 0.001; Southeastern: r = 0.28, p = 0.006; Western: r = 0.22, p = 0.031). However, COVID-19 incidence and SV for *allergic asthma* showed no to low correlations (−0.18 ≤ r ≤ 0.19, 0.038 ≤ p ≤ 0.644, Supplemental Table 2).

### Classification of keywords

The number of keywords for each country differed (Table 1). The 3 search terms per se were searched for most frequently, followed by keywords related to treatment and symptoms. For *asthma*, interest was higher for treatment options (number of keywords n = 64, 30.5%) than for symptoms (n = 23, 11.0%), while the opposite was observed for *bronchial asthma* (treatment: n = 29, 15.8%; symptoms: n = 50, 27.2%). For *asthma* (n = 12, 5.7%) and *allergic asthma* (n = 17, 8.9%), trigger factors were also of substantial interest (Table 2).

**Table 2:** Frequency and percentage of categories for the ten most frequent keywords of the search terms *asthma, allergic asthma,* and *bronchial asthma*. For each country, the top ten keywords were considered if ten were available, otherwise fewer keywords were used.

Category, No. keywords (%)	Asthma	Allergic asthma	Bronchial asthma
general	79 (37.6)	90 (47.1)	74 (40.2)
treatment	64 (30.5)	35 (18.3)	29 (15.8)
symptoms	23 (11.0)	34 (17.8)	50 (27.2)
trigger	12 (5.7)	17 (8.9)	6 (3.3)
attack	10 (4.8)	–	5 (2.7)
children	8 (3.8)	4 (2.1)	7 (3.8)
information	8 (3.8)	3 (1.6)	5 (2.7)
natural/home remedy	6 (2.9)	7 (3.7)	5 (2.7)
cats	–	1 (0.5)	–
progression	–	–	3 (1.6)

No., number of.

**Fig. 4:**
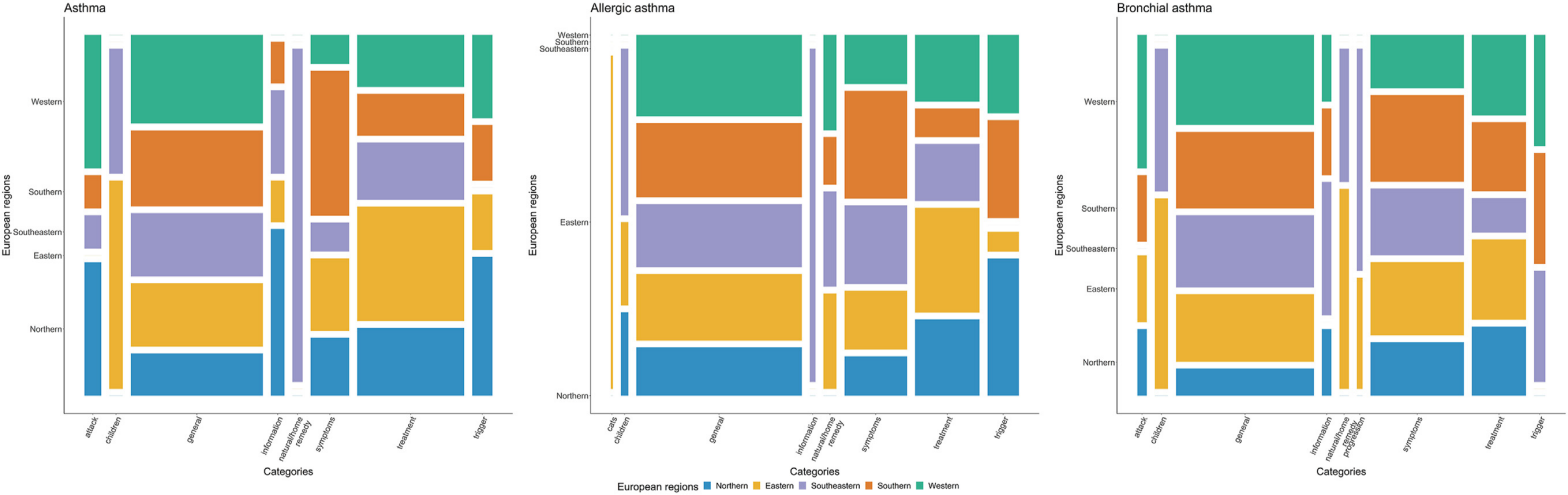
Mosaic plot for the search terms *asthma, allergic asthma,* and *bronchial asthma* for each European region based on the ten most frequent keywords of the corresponding search term. For each country, the top 10 keywords were considered if 10 were available, otherwise fewer keywords were used. European regions were defined geographically as Northern (Denmark, Ireland, Sweden, and United Kingdom), Eastern (Czech, Hungary, Poland, Romania, and Ukraine), Western (Austria, France, Germany, and the Netherlands), Southern (Italy, Malta, Portugal, and Spain), and Southeastern Europe (Bosnia, Croatia, Greece, and Serbia).

Search queries for the search terms in general were relatively equally distributed in all regions except for Northern Europe, where interest was lower. The highest interest in children, information, and natural/home remedies was reported in Eastern and Southeastern regions. A high interest in treatment-related keywords was observed in Eastern Europe. Southern countries were more interested in symptoms and Western countries in attacks and triggers than other regions were (Fig. 4).

## Discussion

The analysis of web search data for *asthma*, *allergic asthma*, and *bronchial asthma* in Europe revealed differences in SV/100 000 inhabitants between the search terms and European regions. Significant seasonal trends were observed for the 3 asthma search terms, with lower SV in European summer months and a higher SV related to *allergic asthma* in springtime. Furthermore, similarities were found between search behavior and Bavarian pollen concentrations and region-specific COVID-19 incidence. People were most interested in the search terms themselves, treatment options, and symptoms, although search behavior varied between European regions.

In this study, the search term *asthma* showed the highest overall SV with 46 104 750 searches, likely because it is the generic term. The lower SV for *bronchial asthma* may be attributed to the fact that this term is not commonly used in all European countries. For example, the WHO International Statistical Classification of Diseases and Related Health Problems 10th revision (ICD-10) code J45 stands for asthma, while in the Swedish and German ICD-10 version, the code J45 stands for bronchial asthma.^33^ The different use of the terms *asthma*, *allergic asthma*, and *bronchial asthma* in the countries studied was also reflected in the different number of related keywords.

The lower SV in Eastern, Southern, and Southeastern Europe may be a consequence of the lower asthma prevalence compared to in Northern and Western regions.^5^^,^^6^^,^^8^ Knowledge about asthma in the general population in Eastern countries, like Ukraine, is limited and stigmatized because “asthma is often believed to be a bad disease, [some] people are afraid of inhalers and refuse to use them”.^34^

Malta showed the highest SV for *asthma*, although it has a disease prevalence of approximately 6%, which is not as high as in other examined countries.^6^ One reason for this may be the high number of tourists, particularly from English-speaking countries, visiting Malta each year. In 2019, about 2.6 million people vacationed in Malta, about 6 times the population of Malta, and approximately 23% of visitors were from the United Kingdom.^35^ This would consequently lead to a higher SV of English queries, as English was the chosen language for Malta in this study.

The study revealed an upward trend in *asthma*-related SV over time and a lower SV in the European summer months, which is in line with the literature regarding prevalence and asthma attacks.^8^^,^^24^^,^^25^ Previously, peaks in asthma exacerbations in spring and winter have been explained by aeroallergens like pollen and viral upper respiratory tract infections, respectively.^24^^,^^25^ Additionally, Canova et al demonstrated evidence of different seasonality of asthma attacks in Northern and Southern Europe, with smaller and later peaks occurring more in Northern Europe than in Southern Europe,^24^ which is consistent with differences in the seasonality of pollen exposure in Europe.^27^ Furthermore, strong parallels between web searches and pollen exposure have been also found in prior studies.^18^^,^^20^ The reason for the higher SV for *bronchial asthma* in winter months may be explained by the higher probability for upper respiratory tract infections during these months,^25^ which is underlined by the negative correlation with grass pollen load.

The study reveals parallels in the temporal trends for pollen concentration and SV, particularly in *allergic asthma*. Combined with pollen concentration data, web search data may therefore support monitoring and prediction of asthma incidence and hospitalizations, as has been proposed and attempted previously.^14^^,^^22^ Further studies are needed to verify this approach. Moreover, climate change is already affecting pollen load, leading to higher or lower prevalence, intensity, and duration of sensitization depending on the type of pollen.^1^^,^^36^^,^^37^ Therefore, crowdsourced data may present a simple and efficient method for tracking the impact of climate change on asthma.

Many parallels have already been observed between COVID-19 incidence and web SV in general^38^ as well as that related to asthma at the beginning of the pandemic in 2020.^21^^,^^30^ The peak in March 2020 for all search terms and regions corresponds to the month in which the WHO declared a global pandemic due to the coronavirus.^38^ In this context, people may have searched for *asthma* and its associated subtypes, as the risk and severity of coronavirus infection is higher in people with preexisting medical conditions.^21^^,^^29^ Another reason may be that an increasing number of individuals searched for asthma information online because they had symptoms such as coughing and shortness of breath.^2^ These symptoms also correspond to symptoms of a COVID-19 infection, suggesting that these symptoms were misinterpreted as asthma symptoms instead.^21^^,^^30^ This idea is further supported by the fact that most searches for bronchial asthma in Southern Europe were recorded in January 2020 before the pandemic officially began. Researchers suspect that by mid-January 2020, the coronavirus had already spread in Northern Italy and consequently further into Europe.^39^ No obvious association was found between the number of COVID-19 cases and SV for *allergic asthma*, which may be attributed to allergic asthma being triggered by exogenous factors.^3^^,^^23^

The categories “natural/home remedies” and “treatment” were of greater interest in Eastern and Southeastern Europe than in other regions, which may be explained by the fear and stigma of asthma in these countries.^34^ Another reason may be poor asthma control and lack of pharmaceutical care and services in these regions.^40^^,^^41^ The high interest in symptoms and treatment across all search terms may indicate an unmet need for disease awareness and disease undertreatment.^10^^,^^42^ Further studies should investigate how barriers to health care access influence asthma outcomes. Moreover, providing reliable information about asthma on the internet should be high priority to raise awareness, improve self-management and thus asthma control.^9^^,^^11^

### Limitations

One limitation is the geographic grouping of the European countries, as a different classification system may have led to significantly different results with other interpretations. Due to the lack of a consistent classification system, this study used that of United Nations (UN), which resulted in 5 European regions of approximately equal size. Including all European countries or other countries may also have yielded different results. Since only the top 10 keywords for each country were used for classification, the frequency of categories may be underestimated. Keywords that did not appear among the top 10 in any country were lost. The inclusion of more keywords would likely have resulted in a more differentiated picture of the public interest in asthma. It should also be noted that only the pollen concentration from Munich was used to analyze the relationship between pollen concentration and SV. Due to regional differences in pollen load and season,^27^ data from other regions may have provided more insight into the role of pollen load on internet search activity. Although pollen seasons show a different phenology between locations, this variability is limited when averaging over 1 month (see www.pollenscience.eu).^43^^,^^44^ No statements on demographic aspects were made in this study, as user information is not provided by Google. However, it can be assumed that older people tend to use the Internet for health information less frequently than younger people.^45^ Another limitation is that only data in the main language of each respective country were considered. Consequently, search queries in other languages were not included, which may have excluded information on the search behavior of non-native speakers like immigrants. In addition, SV may be influenced by news and media, as online searches are strongly driven by media coverage and therefore SV only reflects the overall online interest in the respective search terms not necessarily the epidemiological burden.^46^ It should also be considered that the SV is estimated by the search engine company itself without further options to verify the underlying data.

### Conclusion

Web search analysis has shown to provide insight into the trends and interests related to *asthma*, *allergic asthma*, and *bronchial asthma*. The study emphasized the importance and impact of language, as different search terms were used with varying frequency in different countries. Moreover, the high level of interest in symptoms and treatments for asthma and its subtypes suggests a need for reliable and region-specific education about the disease and for public health campaigns to improve asthma control. Using crowdsourced data and combining multiple data sets appears to be of utmost importance in the era of Big Data to explain patterns, make predictions, and provide a more differentiated picture of the topic.

## Abbreviations

COVID-19 (coronavirus disease), ICD-10 (International Statistical Classification of Diseases and Related Health Problems 10th revision), IQR (interquartile range), r (Spearman correlation coefficient), SV (search volume), SV/100,000 inhabitants (search volume per 100 000 inhabitants), UK (United Kingdom), UN (United Nations), WHO (World Health Organization).

## Funding

This work was supported by the Technical University of Munich, 10.13039/100005481School of Medicine, Munich, Germany.

## Availability of data and materials

The data that support the findings of this study are available from the corresponding author, upon reasonable request.

## Author contribution

Conception and design of the study: LT, SZ.

Acquisition of data: LT, AZ, ES, JE, AD, MB, LS, MS, TT, NB, PC, JB.

Analysis and interpretation: HW, SZ, AZ.

Drafting the article or revising it critically: HW, LT, SZ, AK, CTH, GZ, ES, JE, AD, MB, LS, MS, TT, NB, PC, JB, TB, AZ.

Final approval of the version: All authors agreed to submission and publication of the work.

## Ethics approval

Institutional review board approval and informed consent were not applicable for this study.

## Declaration of competing interest

HW, SZ, AK, CTH, GZ, ES, AD, MB, LS, MS, TT, NB, PC, JB, TB and AZ have no conflicts of interest to declare relating to this work. LT was employed by ViiV Healthcare after the study was conducted. JE has participated in Advisory Board for AstraZeneca.

## References

[1] Agache I., Eguiluz-Gracia I., Cojanu C. (2021). Advances and highlights in asthma in 2021. Allergy.

[2] Dharmage S.C., Perret J.L., Custovic A. (2019). Epidemiology of asthma in children and adults. Front Pediatr.

[3] Celebi Sozener Z., Ozdel Ozturk B., Cerci P. (2022). Epithelial barrier hypothesis: effect of the external exposome on the microbiome and epithelial barriers in allergic disease. Allergy.

[4] Vos T., Lim S.S., Abbafati C. (2020). Global burden of 369 diseases and injuries in 204 countries and territories, 1990–2019: a systematic analysis for the Global Burden of Disease Study 2019. Lancet.

[5] To T., Stanojevic S., Moores G. (2012). Global asthma prevalence in adults: findings from the cross-sectional world health survey. BMC Publ Health.

[6] Statistical Office of the European Union. Persons Reporting a Chronic Disease, by Disease, Sex, Age and Educational Attainment Level [online data code: HLTH_EHIS_CD1E]. Accessed 28 July 2022.

[7] Selroos O., Kupczyk M., Kuna P. (2015). National and regional asthma programmes in Europe. Eur Respir Rev.

[8] Backman H., Räisänen P., Hedman L. (2017). Increased prevalence of allergic asthma from 1996 to 2006 and further to 2016-results from three population surveys. Clin Exp Allergy.

[9] Global Initiative for Asthma (2022). http://www.ginasthma.org.

[10] Holgate S., Bisgaard H., Bjermer L. (2008). The Brussels Declaration: the need for change in asthma management. Eur Respir J.

[11] Price D., Fletcher M., van der Molen T. (2014). Asthma control and management in 8,000 European patients: the REcognise Asthma and LInk to Symptoms and Experience (REALISE) survey. NPJ Prim Care Respir Med.

[12] Bachl M. (2016). Online health information seeking in Europe: do digital divides persist?. SCM.

[13] Ziehfreund S., Tizek L., Zink A. (2022). Websearch-Daten als Gesundheitsdaten? : geografische Unterschiede, zeitliche Trends und Interessenschwerpunkte von Internetsuchmaschinenanfragen in Deutschland. [Web search data as health data? : geographic differences, temporal trends, and topics of interest from internet search engine analyses in Germany]. Hautarzt.

[14] Sousa-Pinto B., Halonen J.I., Antó A. (2021). Prediction of asthma hospitalizations for the common cold using Google trends: infodemiology study. J Med Internet Res.

[15] Verma M., Kishore K., Kumar M., Sondh A.R., Aggarwal G., Kathirvel S. (2018). Google search trends predicting disease outbreaks: an analysis from India. Healthc Inform Res.

[16] Pereira M.P., Ziehfreund S., Rueth M. (2021). Google search trends for itch in Europe: a retrospective longitudinal study. J Eur Acad Dermatol Venereol.

[17] StatCounter GlobalStats (2022). https://gs.statcounter.com/search-engine-market-share/all/europe.

[18] Bousquet J., Onorato G.L., Oliver G. (2019). Google Trends and pollen concentrations in allergy and airway diseases in France. Allergy.

[19] Bousquet J., Agache I., Anto J.M. (2017). Google Trends terms reporting rhinitis and related topics differ in European countries. Allergy.

[20] Karatzas K., Papamanolis L., Katsifarakis N. (2018). Google Trends reflect allergic rhinitis symptoms related to birch and grass pollen seasons. Aerobiologia.

[21] Sousa-Pinto B., Heffler E., Antó A. (2020). Anomalous asthma and chronic obstructive pulmonary disease Google Trends patterns during the COVID-19 pandemic. Clin Transl Allergy.

[22] Sousa-Pinto B., Antó J.M., Sheikh A. (2022). Comparison of epidemiologic surveillance and Google Trends data on asthma and allergic rhinitis in England. Allergy.

[23] Akar-Ghibril N., Casale T., Custovic A., Phipatanakul W. (2020). Allergic endotypes and phenotypes of asthma. J Allergy Clin Immunol Pract.

[24] Canova C., Heinrich J., Anto J.M. (2013). The influence of sensitisation to pollens and moulds on seasonal variations in asthma attacks. Eur Respir J.

[25] Castro C.R., Tarabichi Y., Gunzler D.D., Ayache M. (2019). Seasonal trends in asthma exacerbations: are they the same in asthma subgroups?. Ann Allergy Asthma Immunol.

[26] Biedermann T., Winther L., Till S.J., Panzner P., Knulst A., Valovirta E. (2019). Birch pollen allergy in Europe. Allergy.

[27] Grundström M., Adams-Groom B., Pashley C.H. (2019). Oak pollen seasonality and severity across Europe and modelling the season start using a generalized phenological model. Sci Total Environ.

[28] World Health Organization. WHO Coronavirus (COVID-19) Dashboard: Daily cases and deaths by date reported to WHO. https://covid19.who.int/data. Accessed 27 July 2022.

[29] Song J., Zeng M., Wang H. (2021). Distinct effects of asthma and COPD comorbidity on disease expression and outcome in patients with COVID-19. Allergy.

[30] Barbosa M.T., Morais-Almeida M., Sousa C.S., Bousquet J. (2021). The "Big five" lung diseases in CoViD-19 pandemic - a Google trends analysis. Pulmonology.

[31] Statistical Office of the European Union. Population on 1 January [online data code: TPS00001]. https://ec.europa.eu/eurostat/databrowser/view/tps00001/default/table?lang=en. Accessed 7 July 2022.

[32] United Nations Statistics Division. Methodology - Standard country or area codes for statistical use (M49): Geographic Regions. https://unstats.un.org/unsd/methodology/m49/. Accessed 25 July 2022.

[33] Jetté N., Quan H., Hemmelgarn B. (2010). The development, evolution, and modifications of ICD-10: challenges to the international comparability of morbidity data. Med Care.

[34] Nugmanova D., Sokolova L., Feshchenko Y. (2018). The prevalence, burden and risk factors associated with bronchial asthma in commonwealth of independent states countries (Ukraine, Kazakhstan and Azerbaijan): results of the CORE study. BMC Pulm Med.

[35] National Statistics Office Malta (2020). https://nso.gov.mt/en/News_Releases/Documents/2020/07/News2020_110.pdf.

[36] D'Amato G., Chong-Neto H.J., Monge Ortega O.P. (2020). The effects of climate change on respiratory allergy and asthma induced by pollen and mold allergens. Allergy.

[37] Rojo J., Oteros J., Picornell A. (2021). Effects of future climate change on birch abundance and their pollen load. Global Change Biol.

[38] Schuster B., Tizek L., Schielein M.C. (2021). Aufarbeitung der COVID-19-Pandemie in Deutschland aus Bevölkerungssicht mithilfe von Google Suchanfragen zum Thema “Coronavirus“. [Retracing the COVID-19 Pandemic in Germany from a Public Perspective using Google Search Queries Related to "coronavirus"]. Gesundheitswesen.

[39] Cerqua A., Di Stefano R. (2022). When did coronavirus arrive in Europe?. Stat Methods Appt.

[40] Swieczkowski D., Poniatowski P., Merks P., Jaguszewski M. (2016). The pharmaceutical care in asthma - polish and global perspective. Pneumonol Alergol Pol.

[41] Bumbacea D., Panaitescu C., Bumbacea R.S. (2021).

[42] Winkelmann J., Muench U., Maier C.B. (2020). Time trends in the regional distribution of physicians, nurses and midwives in Europe. BMC Health Serv Res.

[43] Buters J., Prank M., Sofiev M. (2015). Variation of the group 5 grass pollen allergen content of airborne pollen in relation to geographic location and time in season. J Allergy Clin Immunol.

[44] Buters J.T., Thibaudon M., Smith M. (2012). Release of Bet v 1 from birch pollen from 5 European countries. Results from the HIALINE study. Atmos Environ.

[45] Jia X., Pang Y., Liu L.S. (2021). Online health information seeking behavior: a systematic review. Healthcare.

[46] Cervellin G., Comelli I., Lippi G. (2017). Is Google Trends a reliable tool for digital epidemiology? Insights from different clinical settings. J Epidemiol Glob Health.

