# Supplementary material for: Impact of asthma in Europe: A comparison of web search data in 21 European countries
Source: World Allergy Organ J. 2023 Aug 2;16(8):100805. doi: 10.1016/j.waojou.2023.100805 (PMC10410582; doi:10.1016/j.waojou.2023.100805)

Supplemental Material

Supplemental Table 1 Characteristics of the web search volume between 2018 and 2021 for the search terms *asthma, allergic asthma,* and *bronchial asthma* overall, separated by country, region, and season.

	Asthma	Allergic asthma	Bronchial asthma
Overall			
mean (sd)	182.25 (165.96)	10.21 (8.31)	22.96 (28.13)
median [IQR]	121.67 [78.67, 213.56]	8.28 [5.01, 12.99]	10.55 [5.39, 29.68]
***Country**, median [IQR]*			
Austria	183.89 [164.24, 220.8]	19.36 [14.79, 24.3]	65.75 [56.24, 82.49]
Bosnia	61.71 [56.34, 68.8]	7.3 [6.59, 8.3]	9.16 [8.02, 10.31]
Croatia	97.88 [88.19, 107.27]	11.53 [9.57, 13.37]	9.94 [8.28, 11.04]
Czech	65.96 [54.58, 76.83]	1.88 [1.29, 2.46]	1.41 [1.2, 1.78]
Denmark	192.3 [169.99, 209.09]	3.1 [2.41, 4.18]	0.17 [0.17, 0.17]
France	154.36 [135.7, 196.21]	9.19 [7.42, 11.49]	0.67 [0.56, 0.76]
Germany	176.03 [147.45, 213.12]	14.38 [10.34, 21.85]	61.59 [50.67, 76.2]
Greece	100 [88.46, 123.36]	14.17 [11.91, 17.39]	16.69 [13.96, 18.67]
Hungary	108.57 [96.36, 146.71]	6.24 [4.07, 8.03]	8.95 [6.96, 10.26]
Ireland	463.58 [378.19, 566.6]	15.29 [12.44, 17.69]	10.81 [9.07, 12.74]
Italy	90.53 [82.44, 107.88]	10.87 [8.01, 13.34]	26.1 [18.06, 30.23]
Malta	541.98 [480.69, 630.62]	17.22 [12.16, 22.29]	10.13 [8.1, 14.18]
Netherlands	198.88 [163.88, 229.86]	9.11 [7.04, 11.01]	8.07 [7.1, 9.52]
Poland	90.89 [72.87, 101.4]	4.37 [3.59, 6.27]	68.64 [56.51, 79]
Portugal	88.79 [74.81, 110.62]	4.33 [3.36, 5.98]	4.48 [3.7, 5.08]
Romania	104.72 [91.44, 128.46]	7.65 [6.75, 8.72]	83.21 [70, 97.41]
Serbia	58.88 [50.73, 63.36]	4.81 [3.73, 5.74]	10.12 [9.15, 12.39]
Spain	105.34 [85.58, 137.47]	8.11 [6.42, 10.4]	29.42 [23.05, 37.68]
Sweden	276.29 [238.75, 314.17]	13 [8.75, 16.76]	0.49 [0.39, 0.68]
UK	484.87 [404.41, 557.84]	7.88 [6.67, 10.29]	8.33 [6.86, 10.75]
Ukraine	14.99 [11.79, 18.51]	0.1 [0.07, 0.17]	26.18 [20.53, 32.11]

***Region**, median [IQR]*			
Northern	357.91 [314.61, 427.62]	10.53 [7.62, 12.79]	4.43 [3.68, 5.79]
Eastern	89.26 [72.87, 101.4]	4.19 [3.47, 5.65]	26.18 [20.53, 32.11]
Southeastern	78.6 [67.68, 87.68]	9.45 [7.8, 10.72]	10.28 [9.04, 11.92]
Southern	98.76 [82.79, 128.58]	9.24 [7.11, 12.12]	17.69 [13.87, 21.61]
Western	179.97 [155.67, 216.24]	12.59 [9.07, 16.06]	33.93 [29.19, 43.06]
***European seasons**, median [IQR]*			
Spring	123.25 [108.19, 232.91]	11.13 [6.68, 17.18]	10.3 [8.1, 30.52]
Summer	81.95 [68.36, 164.47]	8.72 [4.48, 11.98]	7.54 [4.96, 15.87]
Fall	110.23 [89.2, 193.93]	8.6 [5.31, 11.76]	10.18 [8.37, 30.31]
Winter	116.29 [94.13, 192.41]	7.41 [3.94, 10.06]	10.92 [8.22, 29.98]

European regions were defined geographically as Northern (Denmark, Ireland, Sweden, UK), Eastern (Czech, Hungary, Poland, Romania, Ukraine), Western (Austria, France, Germany, Netherlands), Southern (Italy, Malta, Portugal, Spain), and Southeastern Europe (Bosnia, Croatia, Greece, Serbia).

IQR, interquartile range; sd, standard deviation; UK, United Kingdom.

Supplemental Table 2 Spearman correlations and corresponding p-values between search volume per 100,000 inhabitants for the search terms *asthma, allergic asthma,* and *bronchial asthma*, pollen concentration and COVID-19 incidence. Correlations for COVID-19 incidence were calculated for each European region, while correlations with pollen concentration was considered only for the Western European region.

	**Asthma**	**Allergic asthma**	**Bronchial asthma**	
Pollen concentration	(only Western European regions)			**COVID-19 incidence**
Birch	0.43 (p=0.009)	0.65 (p<0.001)	0.41 (p=0.014)	-0.004 (p=0.969)
Grass	-0.30 (p=0.074)	0.32 (p=0.055)	-0.33 (p=0.048)	-0.537 (p<0.001)
COVID-19 incidence				
Northern	0.31 (p=0.002)	-0.18 (p=0.072)	0.33 (p=0.001)	-
Eastern	0.38 (p<0.001)	0.19 (p=0.038)	0.64 (p<0.001)	-
Southeastern	0.18 (p=0.082)	0.13 (p=0.221)	0.28 (p=0.006)	-
Southern	0.00 (p=0.993)	-0.05 (p=0.644)	-0.18 (p=0.076)	-
Western	0.26 (p=0.010)	-0.07 (p=0.511)	0.22 (p=0.031)	-

European regions were defined geographically as Northern (Denmark, Ireland, Sweden, UK), Eastern (Czech, Hungary, Poland, Romania, Ukraine), Western (Austria, France, Germany, Netherlands), Southern (Italy, Malta, Portugal, Spain), and Southeastern Europe (Bosnia, Croatia, Greece, Serbia). UK, United Kingdom.

Supplemental Figure 1 Country-specific time course of the web search volume per 100,000 inhabitants between 2018 and 2021 in separate graphs for the search terms *asthma, allergic asthma*, and *bronchial asthma*.

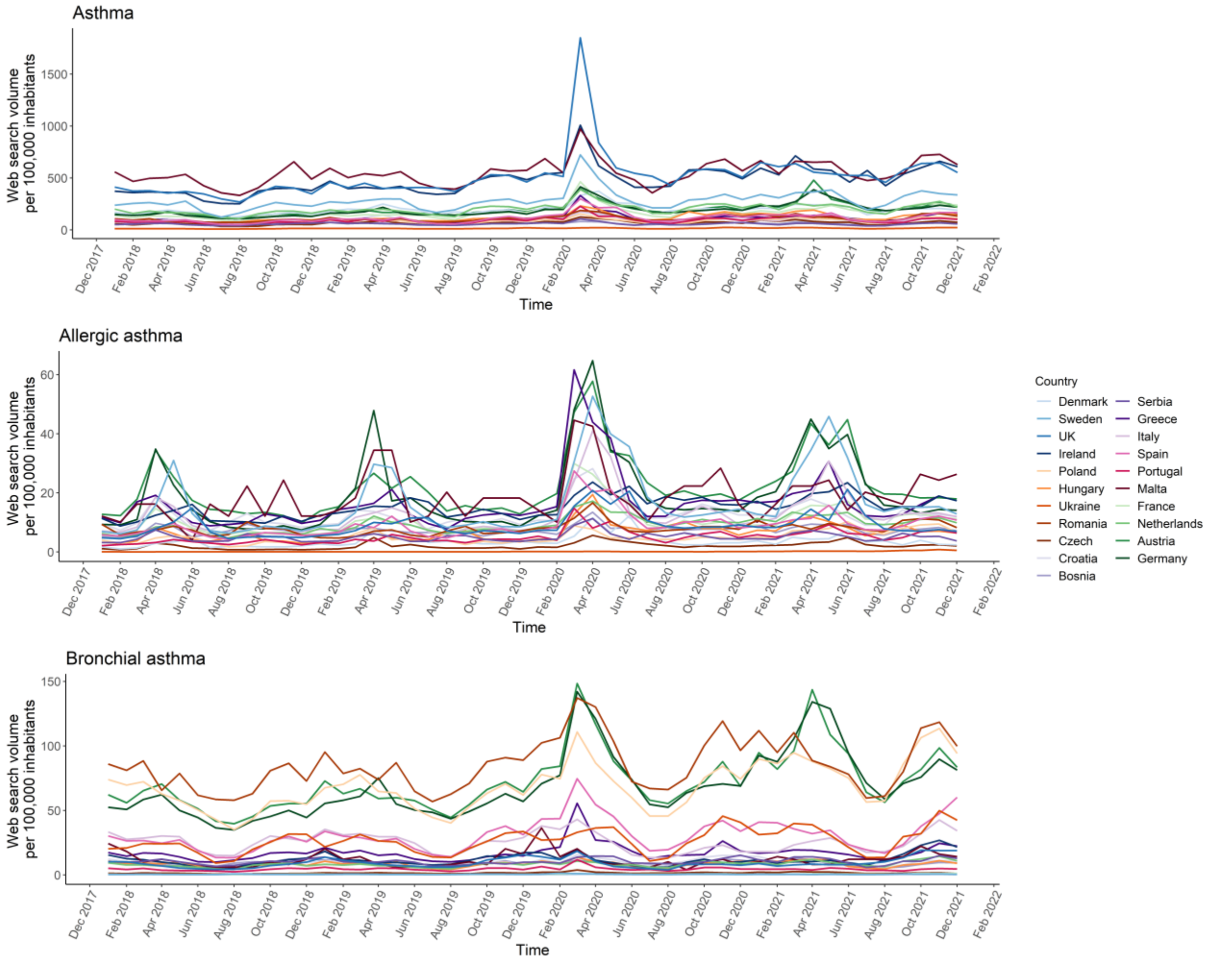

Supplement: Multimedia component 1 [file mmc1.pdf]
